# Long-term Functional Outcomes of Giant Cell Tumours around the Knee treated by Extended Curettage followed by Bone Grafting, Cementation, or a Combination

**DOI:** 10.5704/MOJ.2411.006

**Published:** 2024-11

**Authors:** AQ Khan, Q Raza, MB Abbas, M Chowdhry, MJ Khan

**Affiliations:** Department of Orthopaedic Surgery, Jawaharlal Nehru Medical College and Hospital, Aligarh, India

**Keywords:** giant cell tumour, extended curettage, bone grafting, cementation, PMMA, sandwich technique

## Abstract

**Introduction:**

Recurrence after Giant Cell Tumour (GCT) treatment depends on the type of treatment used. Poly-Methyl-Meth-Acrylate (PMMA) after extended curettage provides structural support and allows for early identification of recurrence but carries a risk of thermal damage to the surrounding healthy tissue. The aim of this study was to compare long-term functional outcomes and complications in patients with GCT around the knee treated with extended curettage and bone grafting or cementation.

**Material and Methods:**

All patients with biopsy-proven GCT, involving either the distal femur or proximal tibia, and treated with either curettage with bone grafting (CBG), curettage with bone cementation (CBC), or curettage combined with grafting and cementation (the Sandwich technique) were included. They were further classified according to Campanacci grading. Patients were followed for a minimum of two years, and all complications were recorded.

**Results:**

The three groups showed a statistically significant difference in terms of persistent pain after surgery (p=0.03), development of long-term arthritis (p=0.01), as well as overall complications (p=0.005). There was no significant difference in terms of the overall recurrence rate between each group (p>0.05). For Campanacci Grade II lesions, there was a statistically significant difference in terms of local recurrence (p=0.01), with lower recurrence rates observed after cementation procedures.

**Conclusion:**

The study indicates that the Sandwich technique was associated with a lower rate of complications compared to CBG or CBC. Patients in the CBG group reported persistent pain, while those in the CBC group exhibited early arthritic changes within five years of the index surgery. Although there was no overall difference in recurrence rates, cementation procedures had a significantly lower rate of recurrence in Campanacci Grade II lesions.

## Introduction

Giant cell tumour (GCT) of bone accounts for approximately 5% of all primary bone tumours^1,2^. A particularly higher incidence has been reported in India and China, where these tumours represent up to 20% of all bone tumours.^1^ These tumours mainly originate from the epiphyseo-metaphyseal, peri-articular regions of long bones and rarely extend into the joint. However, they have been shown to originate from almost any part of the skeleton^2^. These tumours are known to be locally aggressive, with a high likelihood of local recurrence. The recurrence rates of GCT have been reported to vary from 10% to up to 60% in the literature, depending significantly on the type of treatment offered^3-5^. Simple intralesional curettage without the use of any adjuvant agents has been shown to have the highest recurrence rate, while wide marginal excision has the lowest. Recurrence after intralesional curettage is postulated to result from residual tumour cells that are not cleared after mechanical scraping. Therefore, adjuvant therapy is warranted to specifically address this issue^6^. These adjuvants act as cytotoxic agents through their thermal or chemical effects. The chemical adjuvants that have been studied in the literature include alcohol, phenol, hydrogen peroxide, liquid nitrogen, iodine, and cyclophosphamide. Other adjuvants that have been studied include standard cauterisation, argon plasma coagulation, and local radiation. However, these agents have heterogeneous effects on the tumour and show variable success rates, with local recurrence ranging from 5% to 20%^7,8^.

Reconstruction of the bone void has been performed using autogenous grafts, allogenic grafts, or bone cement. Poly-Methyl-Meth-Acrylate (PMMA) is a chemical agent with strong structural properties, as well as thermal and chemical cytotoxic effects^8^. When used as a filler agent in the cavity of GCT, it spreads uniformly and produces a more homogeneous effect on the margins of the tumour, thereby reducing the likelihood of recurrence. Turcotte *et al*^4^ proposed the advantages of the cementation technique combined with curettage. Acrylic cement preserves dynamic stability and aids in the early detection of recurrence through radiological observation of lysis at the cement–bone interface. The cytotoxic properties of the PMMA monomer and the necrotic effects of the heat released during polymerisation help in killing residual tumour cells after extensive curettage. However, the extension of thermal damage into the surrounding healthy tissue poses a risk of destroying subchondral bone and articular cartilage.

The aim of this study was to compare long-term functional outcomes, complications, and recurrence rates in patients with GCT around the knee treated with extended curettage and either bone grafting or cementation alone, as compared to a combination of bone grafting and cementation (the Sandwich technique).

## Materials and Methods

A retrospective study was conducted in the Department of Orthopaedic Surgery, starting from January 2000 to January 2020. This study received IRB approval, and written informed consent was obtained from every patient. The inclusion criteria for the study were skeletally mature patients (age >18 years) with biopsy-proven GCT involving bones around the knee joint (distal femur, proximal tibia) and treated with either curettage with bone grafting (CBG), curettage with bone cementation (CBC), or curettage combined with grafting and cementation (the Sandwich technique). A total of 82 patients who met the inclusion criteria were included in the study (31 in the CBG group, 22 in the CBC group, and 29 in the Sandwich technique group). These tumours were then classified according to histological grade and Campanacci radiological classification^9^. The minimum follow-up period for each patient was two years. The exclusion criteria for the study were skeletally immature patients (age <18 years), involvement of the axial or other appendicular skeleton, incomplete follow-up, or any other treatment modality. A pre-operative Musculoskeletal Tumour Society (MSTS) score was recorded for each patient^10^. The senior author (AQK) performed all surgeries personally.

**Fig. 1:**
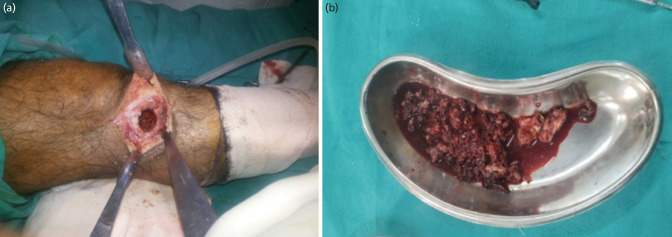
Demonstration of pearls in surgical technique for extended curettage and cementation in treatment of Campanacci Grade I GCT of right proximal tibia (CBG group). (a) Figure showing the cavity after treatment of lesion walls with high-speed burr to break any bony cortical ridges. The curettage was considered complete after the normal cortical bone and cavity were visible. (b) Curetted material was sent for cytopathological and histopathological examination.

For the surgical technique, a third-generation cephalosporin antibiotic was administered 30 minutes prior to the initiation of surgery. The type of anaesthesia was determined by the anaesthesiologist. All patients were operated on in the Operation Theatre (OT) using the Technomed TMI 1206 Hanging Type Orthopaedic OT Table [New Delhi, India] in the supine position. The site of the most thinned-out cortex was localised, and a skin incision was made directly over it. The soft tissue capsule over the tumour was extensively removed. The bone was cut, and a large window was created to scoop out the tumour cells from the lesion. Intralesional curettage was performed using a handheld curette, followed by a high-speed burr to break the bony ridges. The curettage was considered complete only when a normal cortical bone was visualised inside the cavity with punctate bleeding. The curetted material obtained from the cavity was sent for cytological and histopathological examination. This was followed by electrocauterisation for the complete removal of tumour cells. After curettage, the cavity was rinsed with hydrogen peroxide and cleared with pulsed lavage using distilled water (Fig. 1).

Patients were subsequently treated with either curettage with bone grafting (CBG), curettage with cementation (CBC), or curettage combined with cementation and bone grafting (the Sandwich technique). Autogenous cancellous bone grafts were harvested from the iliac crest or ipsilateral fibula. In the groups using bone cement, PMMA was mixed manually until a doughy state was reached, which could be handled by the surgeon. The cavity was then finger-packed with PMMA cement. Cooled saline was used after packing the cavity to irrigate the area around the joint, avoiding thermal damage to the articular cartilage. The wound was closed in layers.

For post-operative evaluation, all patients started physiotherapy on the first post-operative day, with limited or non-weight bearing using a walker or crutches, depending on the location of the tumour. Patients progressed to full weight bearing in both groups at 12 weeks if radiographs showed signs of bone healing. Patients were seen at 2 weeks, 6 weeks, 12 weeks, 6 months, 1 year, and yearly thereafter. At six months and yearly follow-ups, patients were assessed using the MSTS scoring system^10^. Digitised radiographs, including an anterior-posterior (AP) and lateral view of the knee, were taken at each long-term follow-up.

All complications were recorded during the follow-up. Recurrence was detected by studying the bone-cement or bone-bone interface. Each follow-up radiograph was reviewed by a senior radiologist to rule out any possibility of recurrence. Recurrence was diagnosed when there was progressive lysis of more than 5mm at the interface, or if the sclerotic rim at the bone-cement interface was absent. Furthermore, subgroup analysis was performed by classifying the groups according to the Campanacci grade of the tumour and the type of treatment undergone (CBG, CBC, or Sandwich). Kaplan-Meier survival analysis will be performed to assess the long-term survivorship in each treatment group. The recurrence of GCT in each group will be considered a failure.

Statistical analysis of the recorded data was performed using Excel software [Microsoft, Redmond, USA]. The Chi-square test was used to compare nominal variables, with statistical significance set at a level of p<0.05. Fisher’s Exact Test was performed to compare two nominal variables, with statistical significance set at a level of p<0.05.

## Results

**Fig. 2:**
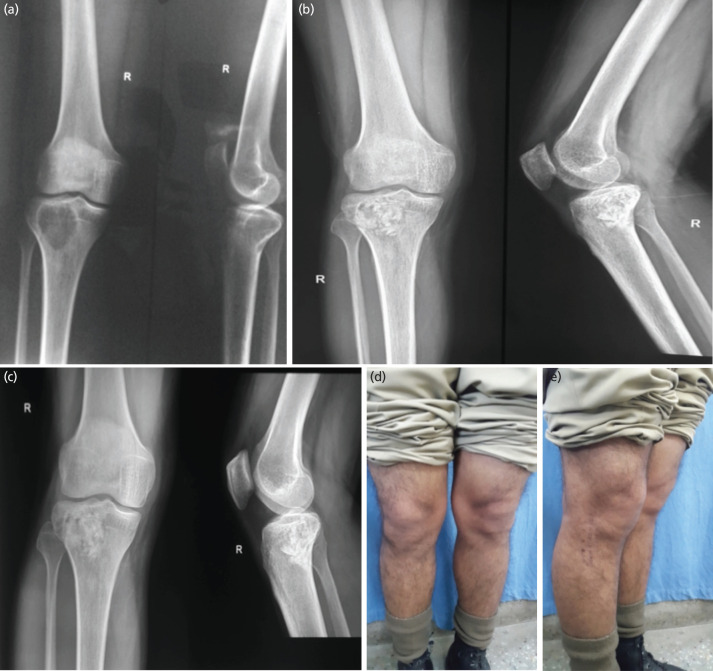
A 32-year-old male with Campanacci grade I GCT of the right proximal tibia operated by curettage and bone grafting (CBG). (a) A pre-operative radiograph (AP/Lateral) of the right knee with proximal tibia showing lytic lesion with a clear surrounding margin of bone present, suggestive of Grade I GCT of the right proximal tibia. (b) Immediate post-operative radiograph of a patient treated with extensive curettage and bone grafting from the contralateral iliac crest. (c) Radiograph at the final two-year follow-up showing a well-healed lesion with no signs of GCT recurrence. (d and e) Clinical photographs of the patient's capabilities at the two-year follow-up showing a full range of motion and complete ability to squat.

In the CBG group (n=31), there were 12 females and 19 males. The average age of the patients was 32.86 ± 3.2 years (range 18 to 58 years). There were 16 cases of Campanacci Grade I lesions, 7 cases of Grade II lesions, and 8 cases of Grade III lesions. All patients were treated by extended curettage and bone grafting (CBG) (Fig. 2). Twenty patients had involvement of the distal femur, and 11 had involvement of the proximal tibia. The mean follow-up was 9.8 years (range 2–18 years). The mean MSTS functional score at the 2-year follow-up was 24.3 (standard deviation (SD) ±3). Sixteen patients (51.6%) developed complications in the CBG group (Table I). There were 9 (29%) cases of persistent pain around the knee joint. Two patients developed superficial infections, which were treated with intravenous antibiotics and daily dressing. One patient developed a deep infection, which required incision and drainage, debridement, removal of the infected graft, followed by staged cementation when the infection markers subsided. Four patients developed subcutaneous hematoma collections, which were drained at the time of three consecutive, daily wound inspections. There were six cases of recurrent GCT (four in Campanacci Grade II and two in Campanacci Grade III tumours) found in this group (Table II). The average time to recurrence was 1.3 years (range 0.9–1.7 years). These patients were treated with extensive curettage and contralateral iliac crest grafting with phenol cauterisation. No patients had subsequent recurrence after five years of follow-up.

**Table I:** A complication seen in each treatment group.

Complications	CBG group (%) N=31	CBC group (%) N=22	Sandwich group (%) N=29	P-value
Persistent Pain	9 (29)	4 (18)	1 (3.4)	0.03
Neuroma	0 (0)	1 (4.5)	0 (0)	>0.05
Infection	3 (9.6)	1 (4.5)	1 (3.4)	>0.05
Hematoma	4 (12.9)	0 (0)	1 (3.4)	>0.05
Arthritis	0 (0)	4 (18)	1 (3.4)	0.01
Total	16 (51.6)	10 (45.5)	4 (13.7)	0.005

**Table II:** Recurrence rate of GCT in each treatment group along with subdivision based on Campanacci Grading. Each subgroup was compared across all treatment groups.

Campanacci Grade	CBG group (%)	CBC group (N)	Sandwich group (N)	p-value
Grade I	0/16 (0)	0/8 (0)	0/10 (0)	>0.05
Grade II	4/7 (57.1)	0/9 (0)	2/13 (15.3)	0.01
Grade III	2/8 (25)	3/5 (60)	1/6 (16.6)	>0.05
Total	6/31 (19.4)	3/22 (13.6)	3/29 (10.3)	>0.05

**Fig. 3:**
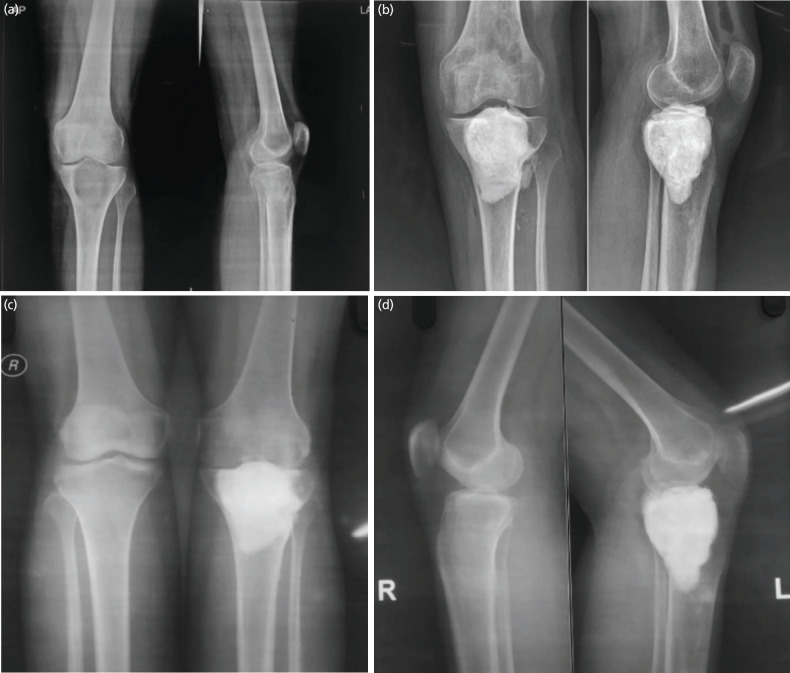
A 27-years old female with Campanacci grade II GCT of left proximal tibia operated with curettage and bone cementation (CBC). (a) Pre-operative radiograph (AP/Lateral) of left knee lytic lesion with expansile lesion having cortical continuity, suggestive of Grade II GCT of left proximal tibia. (b) Immediate post-operative radiograph of patient treated with curettage and bone cementation. (c and d) Radiograph of bilateral knee (AP and Lateral) at final two years follow-up showing well healed lesion with no signs of recurrence of GCT.

In the CBC group (n=22), there were 8 females and 14 males. The average age of the patients was 29.78 ± 3 years (range 18 to 64 years). There were eight cases of Campanacci Grade I lesions, nine cases of Grade II lesions, and five cases of Grade III lesions. All patients were treated by extended curettage and bone cementation (CBC) (Fig. 3). Fourteen patients had involvement of the distal femur, and 8 patients had involvement of the proximal tibia. The mean follow-up was 8.6 years (range 2–14 years). The mean MSTS 93 functional score at 2-year follow-up was 26.3 ± 2. Seven patients (31.8%) developed complications in the CBC group (Table I). There were 4 cases of persistent pain around the knee, which were managed conservatively with painkillers as needed. One patient developed a painful neuroma, which was managed conservatively. One patient had a superficial infection of the suture line, which subsided with oral antibiotics. Four patients complained of persistent joint pain with arthritic changes on radiographs. The mean age of these patients was 48 years (range 44–51 years) at the latest follow-up, and the mean time to radiographic development of arthritis was 5.3 years. All patients developed arthritis in the previously involved compartment of the knee joint (3 medial, 1 lateral). All patients are currently being managed with painkillers. There were 3 cases (13.6%) of local recurrence of GCT, all of which occurred in Campanacci Grade III lesions (Table II). The mean time to recurrence was 1.1 years (range 0.7–1.9 years). All patients were treated with extended curettage and cementation. A second recurrence was observed in one patient, who was subsequently treated with extensive curettage and megaprosthesis application.

**Fig. 4:**
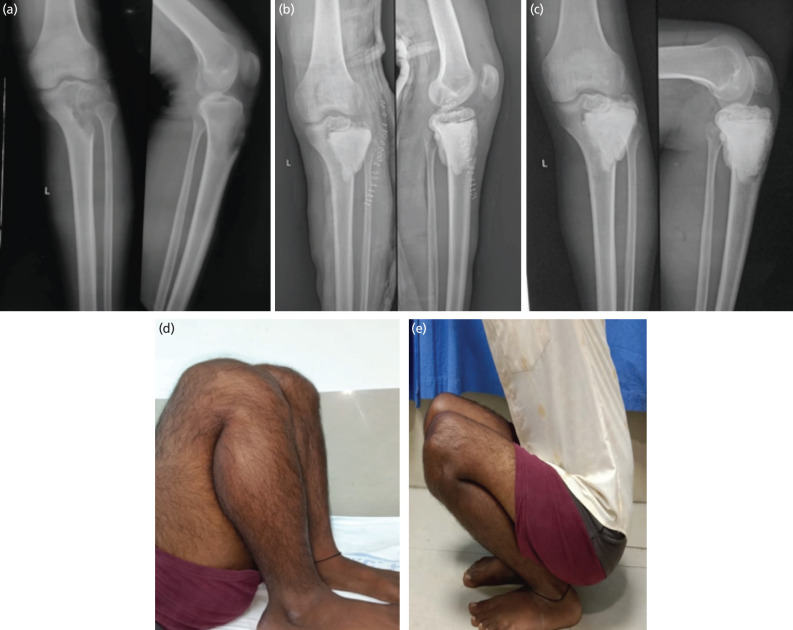
A 20-year-old male with Campanacci grade III GCT of left proximal tibia operated by Sandwich technique. (a) Pre-operative radiograph (AP/Lateral) of left knee with proximal tibia showing lytic lesion extending into intra-articular space with break in cortical continuity, suggestive of Grade III GCT of left proximal tibia. (b) Immediate post-operative radiograph of patient treated with curettage followed by bone grafting and cementation (Sandwich Technique). (c) Radiograph at final two years follow-up showing well healed lesion with no signs of recurrence of GCT. (d and e) Clinical photographs of patient capabilities at two-year follow-up showing full range of motion and complete ability to squat.

In the Sandwich technique group (n=29), there were 17 males and 12 females. The average age of the patients was 30.2 ± 4 years (range 20 to 56 years). There were 10 cases of Campanacci Grade I lesions, 13 cases of Grade II lesions, and 6 cases of Grade III lesions. All patients were treated by extended curettage followed by bone grafting and cementation (Sandwich Technique) (Fig. 4). Nineteen patients had GCT of the proximal tibia, and 10 patients had GCT of the distal femur. The mean follow-up was 8.2 years (range 2.6–10.2 years). The mean MSTS functional score at the 2-year follow-up was 27 ± 2 (Fig. 4). Four patients (13.7%) developed complications in the Sandwich technique group (Table I). There was 1 case of persistent pain in the knee region, managed conservatively with painkillers. One patient developed a hematoma around the distal thigh, which was managed conservatively. There was 1 case of superficial infection, which was treated with intravenous antibiotics and daily dressing changes. There were 3 cases of local recurrences (2 in Campanacci Grade II and 1 in Campanacci Grade III) in this group (Table II). The mean time to recurrence was 0.8 years (range 0.5–1.1 years). All patients were successfully treated with extended curettage followed by bone grafting and cementation.

A comparative analysis between the CBG, CBC, and Sandwich Technique groups showed no statistical difference in age, sex, medical complication rates, patient demographics, and post-operative MSTS scores at two-year follow-up. The three groups showed a statistically significant difference in terms of persistent pain after surgery (p=0.03), development of long-term arthritis (p=0.01), and overall complications (p=0.005). There was no significant difference in terms of the overall recurrence rate between each group (p>0.05). However, for Campanacci Grade II lesions, there was a statistically significant difference found between the three groups in terms of local recurrence (p=0.01), with a significantly lower recurrence after cementation procedures.

**Fig. 5:**
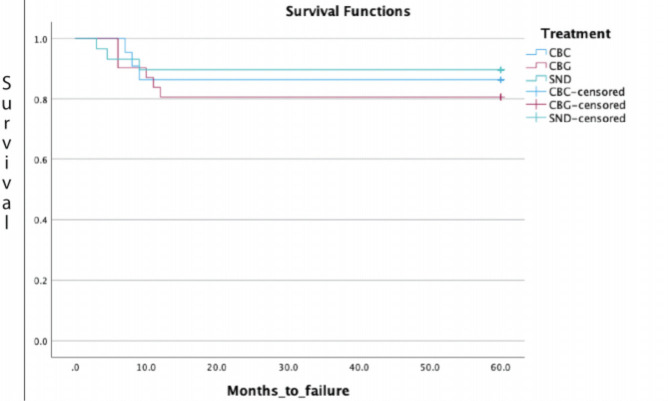
Kaplan-Meier Survival Curve showing recurrence of GCT as failure in each group. The curve reveals that recurrence occurred within 12 months of surgery in each treatment group and there was no late recurrence present in any group.

Fig. 5 shows the Kaplan-Meier Survival Curve displaying the recurrence of GCT as failure in each group. The curve reveals that all recurrences occurred within 12 months of surgery in each treatment group. There were no cases of late recurrence in any treatment group. The three curves were not statistically different from each other in terms of long-term survivorship.

## Discussion

The goal in the treatment of GCT is to balance adequate removal of the tumour with the preservation of limb function. Therefore, the success of treatment is defined both by the rate of recurrence and by post-operative MSTS scores at the final follow-up. In our study, we included tumours around the knee joint, with the distal femur being the most common site, showing a slight predominance in the male population. Since GCTs are locally aggressive tumours, simple curettage has been reported to have a high rate of recurrence, in up to 40% of patients^2,11,12^. Local recurrence of GCT depends on the grade of the tumour, soft tissue extension, and the adequacy of the treatment modality offered. Although wide local excision of the tumour was found to have a significantly low rate of recurrence, it came at the cost of significantly reduced functional outcomes. Campanacci *et al* noted 22% recurrence after intralesional excision, 8% recurrence after marginal excision, and 0% after wide and radical excision^9^. Hence, extended curettage with adjuvant treatment modalities is being studied to manage the tumour locally. These include liquid nitrogen, hydrogen peroxide, phenol, argon laser photocoagulation, bone cement, and bone grafting. In this study, we compared the long-term clinical outcomes of curettage with either bone grafting, cementation, or a combination of both.

In our study, all cases were treated with extensive intralesional curettage with high-speed burr. Algawahmed *et al* showed that the use of intralesional, extended curettage is a crucial step in reducing the chances of local recurrence^13^. After the curettage, the cavity created should be filled with a filler material as the size of the cavity is directly proportional to the chance of developing a complication such as fracture or osteoarthritis. A cavity of greater than 60cc volume is indicated to be filled with a filler material^14^. The cavity has been proposed to be reconstructed using either bone graft, PMMA cement, or calcium phosphate. The primary aim of the filler substance is to provide structural support and prevent any collapse. However, whether the filler substance provides any added advantage in terms of local recurrence is debatable. Becker *et al* reported a recurrence rate of 22% in the cementation group and 49% in the bone grafting group.^11^

A meta-analysis of six studies comparing curettage with either cementation or allogenic grafting concluded a lower recurrence rate with the application of PMMA. This was primarily attributed to the thermal and cytotoxic effects of PMMA on residual tumour cells^15^. However, Turcotte *et al* showed that a filler or an adjuvant does not affect the rate of local recurrence^4^. In our study as well, there was no statistically significant difference between the three groups in terms of local recurrence. Our local recurrence rates were 19.4% in the bone grafting group, 13.6% in the cementation alone group and 10.3% in the combination group (Sandwich technique). This was better than the reported rate in the literature which could be attributed to extensive and thorough curettage along with adjuvant action of PMMA.

In our study, all cases were treated with extensive intralesional curettage using a high-speed burr. Algawahmed *et al* demonstrated that intralesional, extended curettage is a crucial step in reducing the chances of local recurrence^13^. After curettage, the cavity created should be filled with a filler material, as the size of the cavity is directly proportional to the risk of developing complications such as fractures or osteoarthritis. A cavity with a volume greater than 60cc is recommended to be filled with a filler material^14^. The cavity can be reconstructed using either bone graft, PMMA cement, or calcium phosphate. The primary aim of the filler substance is to provide structural support and prevent collapse. However, whether the filler substance provides any added advantage in terms of local recurrence remains debatable. Becker *et al* reported a recurrence rate of 22% in the cementation group and 49% in the bone grafting group^11^.

A meta-analysis of six studies comparing curettage with either cementation or allogenic grafting concluded that PMMA application resulted in a lower recurrence rate. This was primarily attributed to the thermal and cytotoxic effects of PMMA on residual tumour cells^15^. However, Turcotte *et al* showed that the use of a filler or an adjuvant does not affect the rate of local recurrence^4^. In our study, there was no statistically significant difference between the three groups in terms of local recurrence. Our local recurrence rates were 19.4% in the bone grafting group, 13.6% in the cementation-alone group, and 10.3% in the combination group (Sandwich technique). These rates were better than those reported in the literature, which could be attributed to the extensive and thorough curettage combined with the adjuvant action of PMMA.

The advantage of bone grafting is that, if successfully incorporated, the reconstruction becomes permanent. However, early identification of recurrence based on the appearance of a radiolucent line is difficult with bone grafting. Additionally, the use of donor grafts requires a bone bank and carries a risk of allograft rejection. In contrast, the use of PMMA bone cement allows for immediate weight-bearing after surgery and facilitates early detection of recurrence. PMMA also provides direct cytotoxic and thermal effects that help kill residual tumour cells. This effect is particularly evident in Campanacci Grade II lesions of GCT in our study. In the CBC group, there was no recurrence of GCT among Campanacci Grade II lesions. In comparison, there were 4 out of 7 recurrences in the CBG group and 2 out of 13 recurrences in the Sandwich Technique group for Campanacci Grade II lesions. The direct thermal and cytotoxic effects of PMMA are mitigated by the intervening graft in the Sandwich Technique, thereby reducing the adjuvant action of PMMA bone cement. However, among Campanacci Grade III lesions, there was no significant difference in recurrence rates across treatment groups, underscoring the importance of extensive curettage over adjuvant actions for Grade III lesions.

One risk associated with direct cementation of the cavity is thermal necrosis of the subchondral bone and articular cartilage, which can lead to degeneration of the weight-bearing area of the knee joint. It has been suggested that adding a layer of bone graft between the surrounding bone and the cement interface prevents direct transmission of heat, thereby protecting surrounding living tissue. This effect was highlighted in our study. Four patients developed early radiological arthritic changes in the direct cementation group within five years of surgery. In contrast, no patients in the CBG group and only one patient in the Sandwich Technique group developed early arthritis. This difference was statistically significant, indicating that direct cementation has a higher predisposition for early arthritic changes. A study by Benevenia *et al* also found a significantly higher rate of non-oncological complications in the direct cementation group compared to the Sandwich Technique group^16^. However, similar to our study, it found no significant difference in functional outcomes as measured by MSTS scoring.

We acknowledge several limitations of the study. First, it is a retrospective study comparing three different treatment techniques. Second, it includes data from multiple surgeons, and the expertise required for curettage and identification of healthy bone margins is a subjective technical consideration highly dependent on surgeon experience. There is also the potential for surgeon bias, as increased experience with a particular treatment may lead to improved results. Despite these limitations, the study has several strengths. It offers a long-term follow-up with an average duration of around 8-10 years. Furthermore, the inclusion of only patients with GCT around the knee joint results in sufficiently large sample sizes for each group. Future studies would benefit from a randomised controlled trial comparing these treatment groups.

## Conclusion

The results of this study indicate that the Sandwich Technique group was associated with a significantly lower rate of complications compared to the CBG or CBC groups. The CBG group had a significantly higher percentage of patients reporting persistent pain after the procedure. The CBC group had a significantly higher percentage of patients developing early radiological arthritic changes within five years of the index surgery. There was no difference in the overall recurrence of GCT across the three treatment groups. However, for Campanacci Grade II lesions, the CBC group showed a significantly lower rate of recurrence, highlighting the importance of the adjuvant thermal and cytotoxic action of PMMA bone cement.
